# Preoperative nomogram predicting ventriculoperitoneal shunt longevity after initial shunt failure

**DOI:** 10.3389/fneur.2023.1285604

**Published:** 2024-01-12

**Authors:** Dongsheng Liu, Qiheng He, Jianxing Niu, Liangliang Li, Ronghua Geng, Tianqing Cao, Xiaosong Wang, Zeping Lv, Jianghong He, Jizong Zhao, Guoqiang Chen, Yi Yang

**Affiliations:** ^1^Department of Neurosurgery, Civil Aviation General Hospital, Beijing, China; ^2^Department of Neurosurgery, Beijing Tiantan Hospital, Capital Medical University, Beijing, China; ^3^Department of Neurosurgery, National Research Center for Rehabilitation Technical Aids, Beijing, China; ^4^Department of Neurosurgery, Rehabilitation Hospital, National Research Center for Rehabilitation Technical Aids, Beijing, China; ^5^Key Laboratory of Neuro-functional Information and Rehabilitation Engineering of the Ministry of Civil Affairs, Beijing, China

**Keywords:** cox survival regression analysis, preoperative nomogram, VP shunt surgery, VP shunt longevity, initial shunt failure

## Abstract

**Background and objectives:**

Initial shunt failure following ventriculoperitoneal (VP) shunt surgery has a significant impact on the working time of the shunt. However, there are few studies regarding factors affecting VP shunt longevity. Hence, in this study, we aimed to build a nomogram to predict the longevity of the replacement VP shunt in patients with initial shunt failure.

**Methods:**

From 2011 to 2021, 142 patients with initial VP failure who underwent VP shunt revision were enrolled and relevant clinical and demographic factors were analyzed. Univariate and multivariate Cox proportional hazard regression models were used to choose predictors, and a nomogram was constructed using nine independent prognostic variables: sex, age, hydrocephalus type, intensive care unit admission, tracheostomy, decompressive craniectomy, craniotomy, lumbar cisterna drainage, and ventricular drainage. The prediction models’ discrimination, accuracy, calibration, and clinical value were evaluated using Harrell’s C-index, a calibration plot, and decision curve analysis.

**Results:**

At 1 month, 3 months, and 5 years, the nomogram’s C-index was 0.680, 0.708, and 0.694, respectively. The nomogram’s calibration plot provided a good fit for the overall prediction over the course of 1 year. Decision curve analysis predicted that 1–3 months after surgery will yield good net benefits between 30 and 50% probability thresholds.

**Conclusion:**

A preoperative nomogram may be an effective tool for assessing VP shunt longevity after initial VP shunt placement.

## 1 Introduction

Approximately 30,000 ventriculoperitoneal (VP) shunt placements are performed annually in the United States (1), but complications from the surgery remain high. With the improvement in treatments for severe brain injury, more patients with secondary hydrocephalus (hydrocephalus with an identifiable cause) are being treated, and the number of shunt operations is increasing. In both pediatric and adult populations, shunt blockage and infection are the most common causes of shunt failure (2, 3), with infections causing early failures and catheter blockage causing late failures (4). Shunt catheter obstruction, a shunt blockage caused by debris, such as blood, protein fluid, or choroid plexus, is the most common cause of shunt malfunction; yet, the factors that contribute to this problem remain elusive. Obstruction can occur in the proximal catheter, within the valve, or within the distal catheter, and the most common site of obstruction is the proximal catheter (5–7). Infection is the second most common cause of shunt malfunction (2, 3), with a reported rate of approximately 8–15% among patients who undergo VP shunt placement (8–11). Intracranial infection is characterized by fever, positive meningeal signs, vital sign abnormalities, and positive cerebrospinal fluid (CSF) bacterial cultures. Risk factors for shunt infection include young age (4, 9–12), postoperative CSF leakage (10), glove holes during shunt handling (10), African American race (9), public insurance (9), previous shunt infections, and etiology of intraventricular hemorrhage (9, 12). Paff and Reddy identified risk factors for shunt failure, including age, causes and types of hydrocephalus, surgical aseptic technique, and shunt pump improvement (2). Despite improvements in the shunt system, valve design, and sterile practices, the rate of shunt malfunction has not decreased in recent decades (13).

Initial shunt removal surgery is performed to adjust the original shunt after shunt failure has occurred. VP shunt longevity refers to the time interval between the initial VP shunt surgery and the initial shunt removal surgery. Gonzalez et al. reported multiple shunt failures after the initial or revision surgery in children (14). Hence, improving VP shunt longevity would reduce the burden on medical resources. Despite many studies on intraoperative procedures, the effect of risk factors such as other surgical procedures and shunt preparation environment on VP shunt longevity is poorly reported. We analyzed the clinical data of patients with initial VP shunt failure to determine the factors affecting VP shunt longevity after revision. Finally, a nomogram was constructed to predict VP shunt longevity after initial VP shunt failure.

## 2 Methods

### 2.1 Patients and data collection

From 1 February 2011 to 1 February 2021, 180 patients who underwent initial shunt revision at our hospital were recruited. Cases with lumbar cisternae peritoneal shunt, incomplete data, and revision abandonment were excluded. Informed consent was obtained from the recruited patients. Details of sociodemographic factors, such as sex and age, as well as other factors such as causes of hydrocephalus, causes of VP shunt failure, and history before the initial VP (intensive care unit [ICU] admission, tracheostomy, decompressive craniectomy, craniotomy, lumbar cisterna drainage, and ventricular drainage) were collected. The longevity (months) of the initial or revision VP shunt was calculated. We followed the Strengthening Reporting on Observational Studies in Epidemiology (STROBE) guidelines to improve the quality of the study. The study was approved by the Institutional Review Board of the Beijing Tiantan Hospital (KY202207302).

### 2.2 Statistical analysis

All data were analyzed using SPSS version 26 (SPSS Inc., Chicago, Illinois, United States). Values are expressed as *n* (%), x̅ ± s, or M (Q1–Q3). The Kaplan–Meier survival analysis was compared between groups and used as a survival analysis function curve, and a value of *p* <0.05 was considered statistically significant. The multivariate analysis was carried out in the Cox regression model of each factor, and a value of *p* <0.05 was considered to be statistically significant. R 3.1.2 (The R Foundation for Statistical Computing, Vienna, Austria) with the RMS statistical package was used to build a nomogram based on the Cox regression model. R 3.1.2 software was used for the ROC curve, calibration curves, and decision curve analysis.

## 3 Results

**Figure 1:**
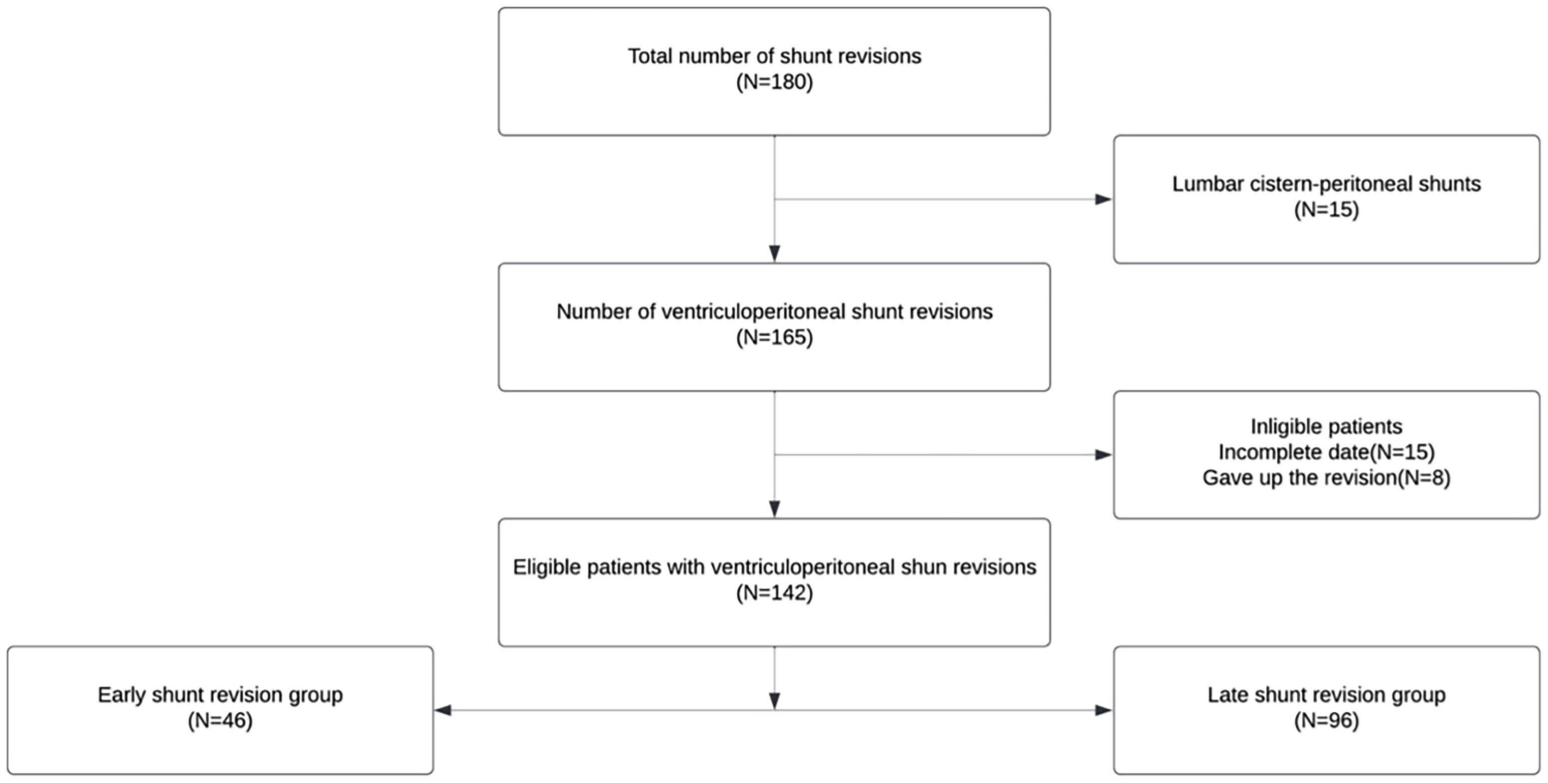
Flowchart of the study.

From 1 February 2011 to 1 February 2021, 142 patients who met the inclusion criteria were included in the analysis (Figure 1).

Among these patients, 95 (66.9%) were men and 47 (33.1%) were women, with a mean age of 35.22 ± 18.61 years. All patients included in this study had supratentorial hydrocephalus. The Kaplan–Meier survival analysis showed no difference in VP shunt longevity between sexes and between age groups (Table 1).

**Table 1:** Kaplan–Meier analysis of demographic factors and shunt survival time (months).

Demographic characteristics		*n* (%)		*M*	*Q1*	*Q3*	*p*-value
Sex	Female	47	(33.1%)	5.20	0.50	36.60	0.392
	Male	95	(66.9%)	2.60	0.50	41.50	
Age	Minors	27	(19.0%)	4.60	1.10	13.10	0.316
	Adults	115	(81.0%)	2.80	0.80	19.60	

The overall VP shunt longevity was 3.1 (0.88–18.48) months, ranging from 0.10 to 352.80. Of the patients with primary hydrocephalus (hydrocephalus with no apparent cause), 36.6% (52/142) had a median VP shunt longevity of 10.45 (0.50, 60.9) months and 63.4% (90/142) patients with secondary hydrocephalus had a median VP shunt longevity of 1.95 (0.60, 18.10) months. Secondary hydrocephalus cases included 38 (42.2%) cases of brain hemorrhage, 33 (36.7%) of brain trauma, 14 (15.6%) of brain tumors, and 5 (5.6%) of other causes. Survival analysis showed a statistically significant difference between primary and secondary hydrocephalus (*p* < 0.05). In all 142 cases of shunt failure, there were 70 cases (49.3%) of shunt infection with a median longevity of 1.15 (0.60, 5.80) months and 72 cases (50.7%) of shunt blockage with a median longevity of 5.35 (2.15, 39.05) months. The Kaplan–Meier survival analysis showed a difference between the infection and shunt blockage groups (*p* < 0.05). The analysis detected six clinical factors affecting VP shunt longevity: ICU admission, tracheostomy, decompressive craniectomy, craniotomy, lumbar cisterna drainage, and ventricular drainage. The median time of each group is listed in Table 2.

**Table 2:** Kaplan–Meier survival analysis of shunt by clinical factors (months).

Clinical factors		*n*	(%)	*M*	*Q1*	*Q3*	*P*-value
Median primary shunt survival time		142	100%	3.1	0.88	18.48	
Hydrocephalus causes	Primary hydrocephalus	52	(36.6%)	10.45	0.50	60.90	0.002^*^
	Secondary hydrocephalus	90	(63.4%)	1.95	0.60	18.10	
Secondary hydrocephalus	hemorrhage	38	(42.2%)	1.25	0.60	5.00	0.154
	Trauma	33	(36.7%)	2.80	0.90	5.80	
	Brain tumor	14	(15.6%)	2.85	0.90	32.60	
	Others	5	(5.6%)	2.60	0.80	73.10	
VP failure cause	Blockage	72	(50.7%)	5.35	2.15	39.05	0.000^*^
	Infection	70	(49.3%)	1.15	0.60	5.80	
ICU admission	Yes	66	(47.1%)	1.80	0.50	9.70	0.000^*^
	No	74	(52.9%)	5.70	0.70	60.80	
Tracheotomy	Yes	54	(38.0%)	1.75	0.50	18.10	0.000^*^
	No	88	(62.0%)	5.25	0.80	56.00	
Decompressive craniectomy	Yes	50	(35.2%)	1.05	0.40	18.10	0.000^*^
	No	92	(64.8%)	5.20	0.80	49.00	
Craniotomy	Yes	77	(54.2%)	1.90	0.50	18.10	0.002^*^
	No	65	(45.8%)	7.70	0.70	60.80	
Lumbar cisterna drainage	Yes	40	(28.2%)	1.15	0.40	13.10	0.001^*^
	No	102	(71.8%)	5.00	0.80	44.70	
External ventricular drainage	Yes	37	(26.1%)	1.90	0.40	36.80	0.025^*^
	No	105	(73.9%)	4.60	0.70	40.10	

^*^Indicates statistically significant difference.

**Figure 2:**
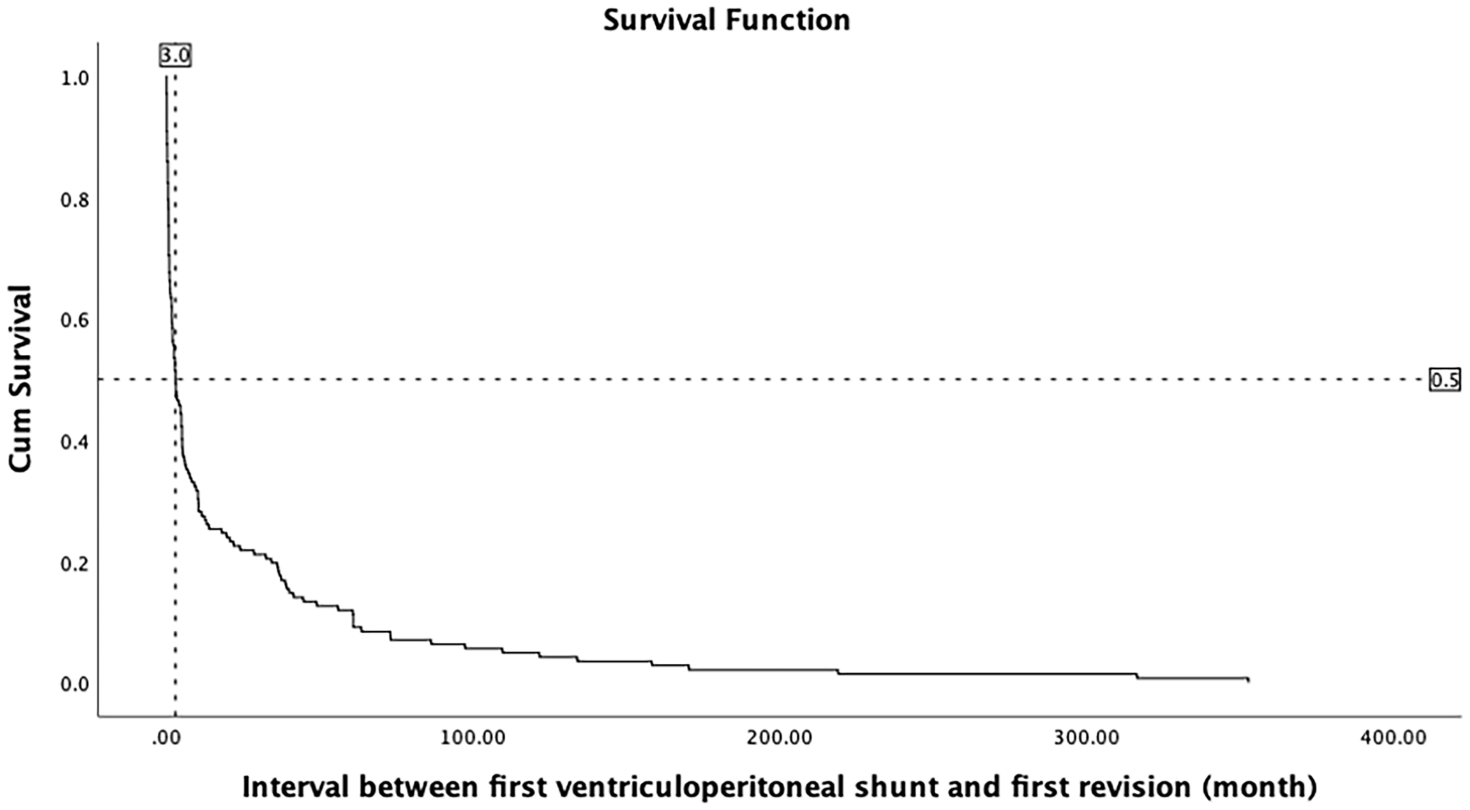
Survival curve shows that the median VP shunt longevity in 142 failed cases was 3 months.

The causes of hydrocephalus, causes of shunt failure, and perioperative risk factors were grouped. The Kaplan–Meier survival analysis was performed between groups, and the results were statistically different (*p* < 0.05, Supplementary Figures S1–S5). The survival curve shows that the median initial VP shunt longevity in 142 failed cases was 3 months (Figure 2).

We built a Cox model for clinical variables affecting shunt longevity. Infections after VP placement and lumbar cisterna drainage before VP placement were risk factors for shunt survival (Table 3).

**Table 3:** Multivariate COX regression analyzed VP shunt survival time.

Clinical factors	*P*-value	OR	95%CI	
ICU admission (yes vs. no)	0.706	1.143	0.571	2.288
Tracheotomy (yes vs. no)	0.611	0.821	0.385	1.755
Decompressive craniectomy (yes vs. no)	0.160	1.574	0.836	2.963
Lumbar cisterna drainage (yes vs. no)	0.021^*^	1.612	1.075	2.415
External ventricular drainage (yes vs. no)	0.367	1.217	0.794	1.864
Age (adult vs. minors)	0.452	0.833	0.517	1.341
Causes of hydrocephalus (secondary vs. primary)	0.471	1.212	0.718	2.046
Causes of shunt failure (infection vs. blockage)	0.001^*^	1.862	1.294	2.678

^*^Indicates statistically significant difference.

VP, ventriculoperitoneal; ICU, intensivecare unit; vs, versus.

**Figure 3:**
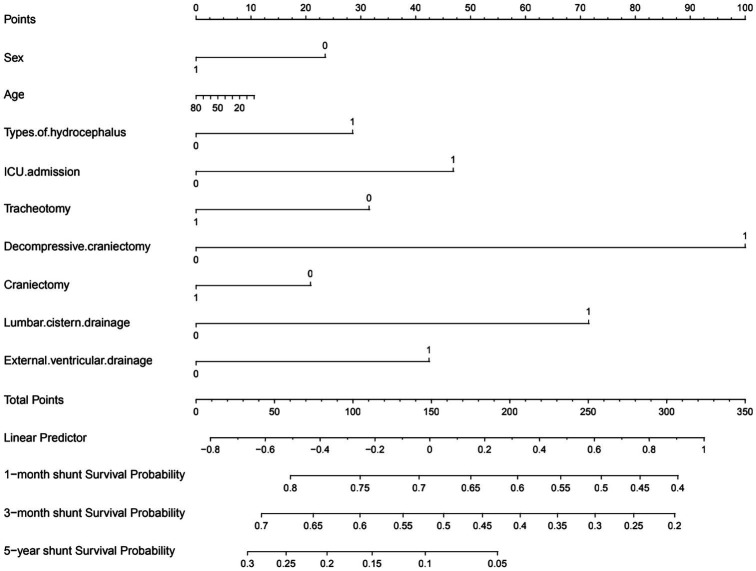
Preoperative nomogram estimating the 1-month to 5-year shunt survival rate after initial VP shunt (in the table, yes answers were assigned 1 and no answers were assigned 0).

**Figure 4:**
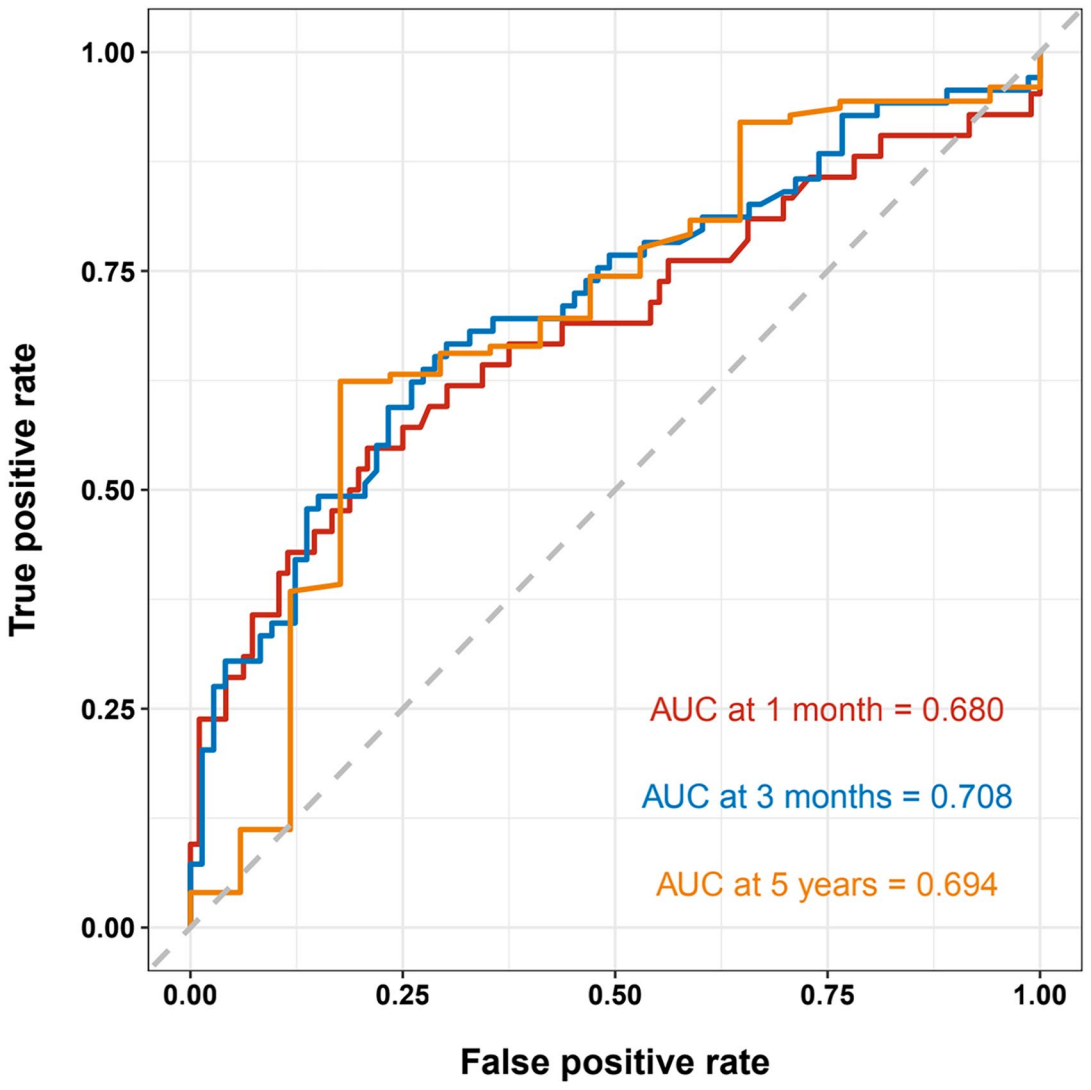
Receiver operating characteristic curve shows the sensitivity and specificity of a shunt survival test. At 1 month, 3 months, and 5 years, the nomogram’s area under the curve was 0.689, 0.708, 0.694, respectively.

**Figure 5:**
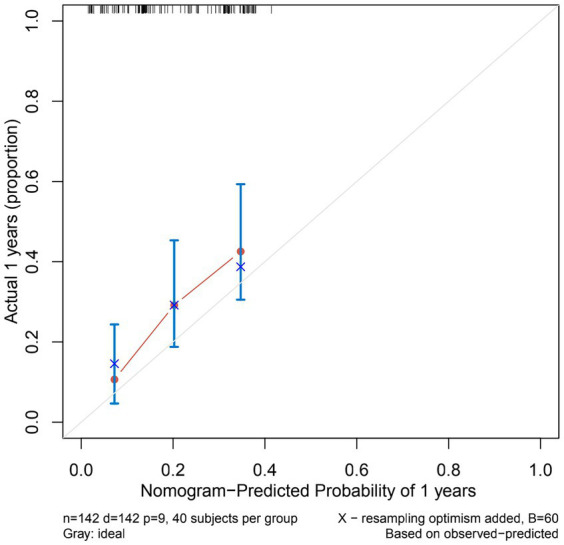
Calibration curves of the nomogram predicting VP shunt longevity in 1 year. The *Y*-axis of calibration curves reflects actual probability, the *X*-axis predicts probability, and the diagonal dashed line represents a flawless model’s prediction. Our calibration plots are close to the ideal line.

The nomogram showed that a shunt with a score of 260 has a 30-day survival rate of 50%, a shunt with a score of 160 has a 90-day survival rate of 50%, and a shunt with a score of 35 has a 5-year survival rate of 30% (Figure 3). At 1 month, 3 months, and 5 years, the nomogram’s area under the receiver operating characteristic curve was almost 0.7 (Figure 4). Calibration curves showed that the nomogram has a good predictive value (Figure 5).

**Figure 6:**
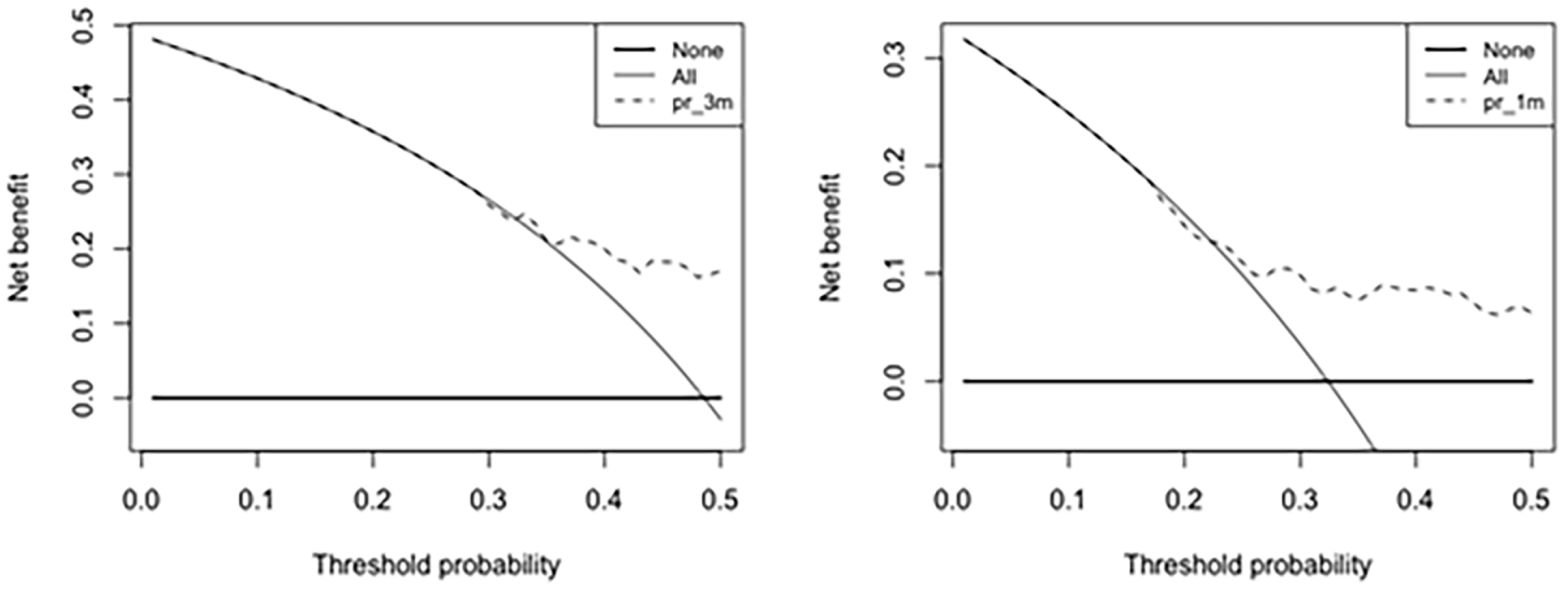
Decision curve analysis of the nomogram predicting VP shunt longevity in 1 month and 3 months. The *Y*-axis reflects net benefit, and the *X*-axis reflects threshold probability. Net benefit rate is the dotted line above the solid line.

Decision curve analysis of the nomogram predicting VP shunt longevity was performed at 1 month and 3 months. In the nomogram in decision curve analysis, a prediction of 1–3 months after surgery yielded good net benefit, with probability thresholds between 30 and 50% (Figure 6).

## 4 Discussion

In this study, we found that lumbar cisterna drainage and shunt failure due to infection are significantly associated with shorter shunt longevity. We also generated a nomogram including age, sex, type of hydrocephalus, ICU admission, tracheotomy, decompressive craniectomy, craniectomy, lumbar cisterna drainage, and external ventricular drainage and found it to be a useful predictive tool. To the best of our knowledge, this is a novel study focused on patients with initial shunt failure.

The most common surgical treatment for hydrocephalus is VP shunting, but surgical complications remain high. Paff et al. reported that most VP shunting complications occur in the first postoperative year, with a failure rate of 11–25%. Despite advances in neurosurgery, VP shunt removal rates are significantly high and require surgical replacement. Our study indicated that age did not affect shunt longevity, whereas Kesava and Yvonne et al. observed that pediatric patients may require more frequent removal. Further studies are required to confirm this action in pediatric patients.

In our study, almost 50% of shunt revisions happened within 3 months. Shunt longevity rate decreases sharply within 3 months of surgery and then increases slowly, indicating that there is a high risk of early failure after shunting. The incidence of late shunt failure decreases year by year, so clinical prevention and treatment of shunt failure should focus on the causes of early failure. Hosainey et al. reported 90 days following shunt surgery as the early failure cutoff (15). Anderson et al. reported 30 days after early shunt failure as a standard (8). Our findings are consistent with these two studies, demonstrating that it is feasible to consider 3 months after the initial ventricular shunt as a reference time to study early shunt failure. Our preoperative nomogram used 30 and 90 days as predictors, based on the median VP shunt longevity.

We also found a significantly different shunt longevity between cases of primary and secondary hydrocephalus. Patients with primary hydrocephalus have no clear lesions and are less likely to undergo surgery after shunting, which is helpful in prolonging shunt longevity. In contrast, patients with secondary hydrocephalus have a clear lesion, which may affect shunt surgery, and there is more opportunity for other procedures to be performed, which ultimately affects shunt survival.

Infection causes most early VP shunt failures. Late shunt failure is usually caused by blockage. Both blockage and infection were major contributors to shunt failure in this study. In contrast to the blocked group, the infected group had a shorter median and mean VP shunt longevity. Shunt failure caused by infection is more likely to be associated with shorter shunt longevity, and post-infection time is related to early VP shunt failure. Our data showed that the median VP shunt longevity for shunt infection was 1.15 (0.60, 5.80) months and that the longevity for shunt obstruction was 5.35 (2.15, 39.5) months. Conen et al. reported that most shunt infections occur within 1 month of VP shunting (16), and Test et al. reported that the median time to infection from shunt surgery is 20–23 days (17). We presume that 50% of shunt infections arise within 1 month of shunting, and infection significantly affects shunt survival. ICU admission may be an environmental factor involved in infection. In contrast, tracheostomy could improve pneumonia and systemic inflammatory conditions.

Decompressive craniectomy is the major treatment method for head trauma; however, additional risks cannot be neglected (18). Intracranial hemorrhage and wound infections increase the risk of VP shunt failure in patients with head trauma or intracerebral hemorrhage undergoing decompressive craniectomy. The duration from shunt placement to failure after decompressive craniectomy surgery is 4 months (18). Early shunt failure may be related to the high failure rate after decompressive craniectomy, and our results are consistent with those of previous studies. The nomogram shows that decompressive craniectomy remains a high risk and should be carefully evaluated.

Neurosurgeons often use lumbar cisterna drainage. Lumbar cisterna drainage is implemented for cases of secondary hydrocephalus, such as acute hydrocephalus, hydrocephalus after cerebral hemorrhage, hydrocephalus after a brain tumor, and infectious hydrocephalus, to control intracranial pressure; decontaminate, retain, and test CSF; and play a temporary drainage role for VP shunt. Some studies claim that the rate of lumbar cisterna infection is over 40% (2, 9, 19–22). Chen et al. reported that lumbar cisterna drainage is a risk factor for intracranial infection (9, 23). Independent risk factors for lumbar cisterna drainage and intracranial infection include site leakage, drainage tube blockage, and duration of continuous drainage (20). Long-term lumbar cisterna drainage may cause infection and shunt failure. VP shunting can only be conducted once CSF indicators meet the shunt criteria, which involves prolonged clearing and many tests. Hoefnagel et al. indicated that CSF sampling frequency is a risk factor for ventricular drainage infection (24). Rogier et al. reported that long-term lumbar cisterna draining is a risk factor for intracranial infection (20), and repeated CSF testing increases intracranial infection risk.

CSF testing is necessary before VP shunting, but it also has a certain false positive rate. Dorresteijn et al. reported that clinical factors and biochemical and microbiological markers have limited diagnostic value in differentiating ventriculitis and aseptic inflammation in patients with CSF external drainage (25). Pfisterer et al. indicate that ventricular drainage limits the efficacy of CSF investigation in diagnosing bacterial meningitis (26). Routine CSF biochemistry, Gram stain, inflammatory factor indicators, bacterial culture, and PCR detection may be unclear in detecting true intracranial infections, which are hidden threats to VP. Intraventricular drainage tube susceptibility and CSF tests may miss true infection, and uncured infection may recur after shunting, resulting in VP failure. Our study found that the external ventricular drainage group had a shorter VP shunt longevity than the group without surgery, which may be supported by the above findings. Ventricular drainage and lumbar cisterna drainage before the VP shunt placement were substantially related to infection, and our predictive models showed that these causes were important shunt longevity factors.

Nomograms are commonly used as a diagnostic tool to forecast the likelihood that a patient might suffer from an illness. To the best of our knowledge, our study is the first to model the longevity of VP shunts. Few researchers have utilized clinical and demographic factors to create risk factor prediction models for VP shunts. The majority of prior research focusing on risk factors for VP shunt failure was based on small sample size VP shunt failure cohorts, which may not accurately predict the longevity of VP shunts. Our nomogram was based on 142 cases of VP shunt failure, and this large sample size ensured the reliability and generalizability of the results. At 3 months, the C-index of our nomogram was 0.708, indicating that the discriminatory power of our nomogram was useful for predicting early VP shunt failure. In addition, the C-index of the nomogram of the 5-year prediction model was 0.694, showing that this nomogram is also useful for predicting late VP shunt failure. The majority of our findings indicate the nomogram has excellent calibration, and our calibration plots are close to the perfect line.

Through the decision curve analysis, our study found that the nomogram has better short-term predictive power than long-term ability. Early prediction at 1–3 months after surgery yielded good net benefit, with the probability thresholds between 30 and 50%. Short-term predictions are more clinically realistic than long-term predictions because our study found that 50% of VP shunts are removed within 3 months after their placement. Hence, if the probability of longevity of the shunt can be predicted before shunting, the advantages and disadvantages can be considered before surgery to choose temporary drainage and wait until the internal environment and CSF environment are stable before the VP shunt placement, which can reduce the rate of postoperative shunt tube revision surgery. We believe that this preoperative nomogram may be an effective tool for assessing VP shunt longevity after the initial VP shunt.

This study’s retrospective essence has certain limitations. Only cases with complete data were included, which may have induced bias. This study included fewer pediatric cases as only 19% of the included patients were children, which may limit the usefulness of the nomogram as a generalized tool for the pediatric population. More studies are required to validate our findings. Due to the lack of an external database, we were unable to perform external verification, which may affect the reliability of the nomogram.

## Data availability statement

The raw data supporting the conclusions of this article will be made available by the authors, without undue reservation.

## Ethics statement

The studies involving humans were approved by the institutional review board of Beijing Tiantan Hospital. The studies were conducted in accordance with the local legislation and institutional requirements. Written informed consent for participation was not required from the participants or the participants’ legal guardians/next of kin because this was a retrospective study from an established cohort and thus separate informed consent was not required.

## Author contributions

DL: Conceptualization, Data curation, Formal analysis, Investigation, Methodology, Writing – original draft. QH: Project administration, Writing – review & editing. JN: Data curation, Writing – review & editing. LL: Data curation, Writing – review & editing. RG: Data curation, Writing – review & editing. TC: Data curation, Writing – review & editing. XW: Writing – review & editing. ZL: Conceptualization, Writing – review & editing. JH: Supervision, Writing – review & editing. JZ: Writing – review & editing, Supervision. GC: Supervision, Writing – review & editing. YY: Conceptualization, Supervision, Writing – original draft, Writing – review & editing.
