# Supplementary material for: Preoperative nomogram predicting ventriculoperitoneal shunt longevity after initial shunt failure
Source: Front Neurol. 2024 Jan 12;14:1285604. doi: 10.3389/fneur.2023.1285604 (PMC10811779; doi:10.3389/fneur.2023.1285604)
Supplement: Supplementary file 1 [file Data_Sheet_1.docx]

Supplementary Material

Preoperative nomogram predicting the ventriculoperitoneal shunt longevity probability after initial shunt failure: based on 142 ventriculoperitoneal shunt failure cases

# Supplementary Figures and Tables

## Supplementary Figures


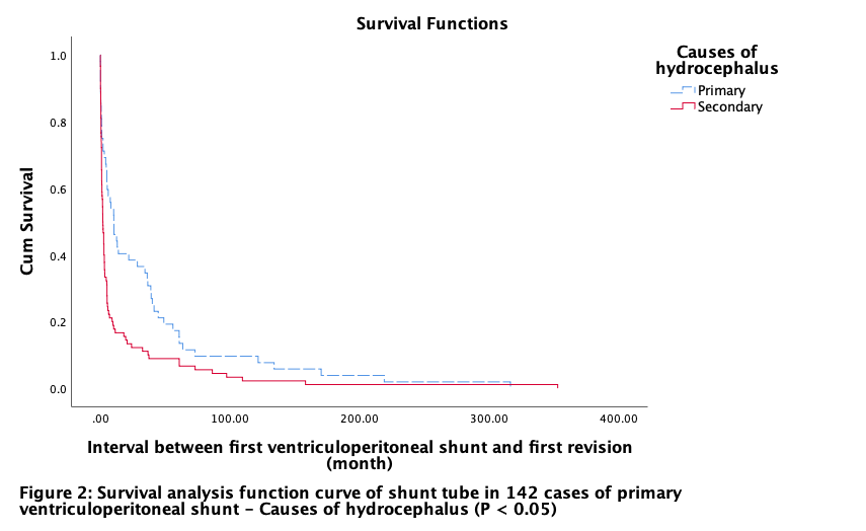


**Supplementary Figure 1.** The cause of hydrocephalus were grouped and analyzed by Kaplan-Meier survival (P < 0.05).


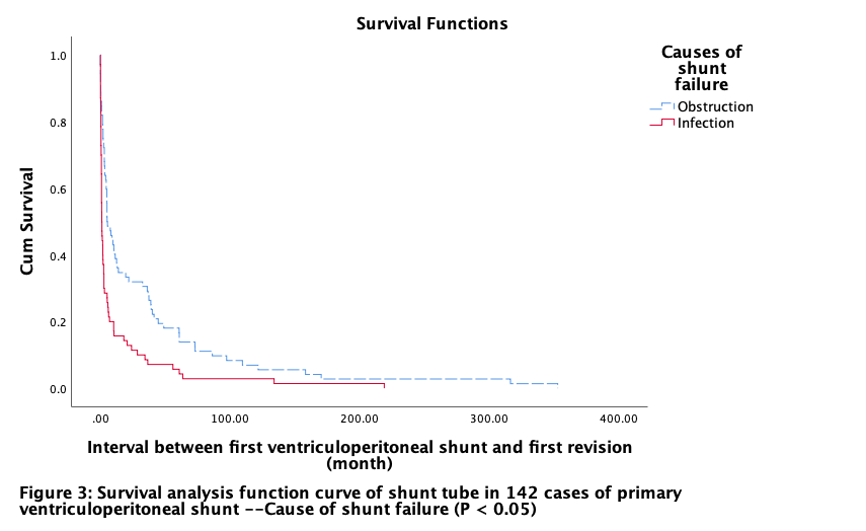


**Supplementary Figure 2.** The cause of shunt failure were grouped and analyzed by Kaplan-Meier survival (P < 0.05).


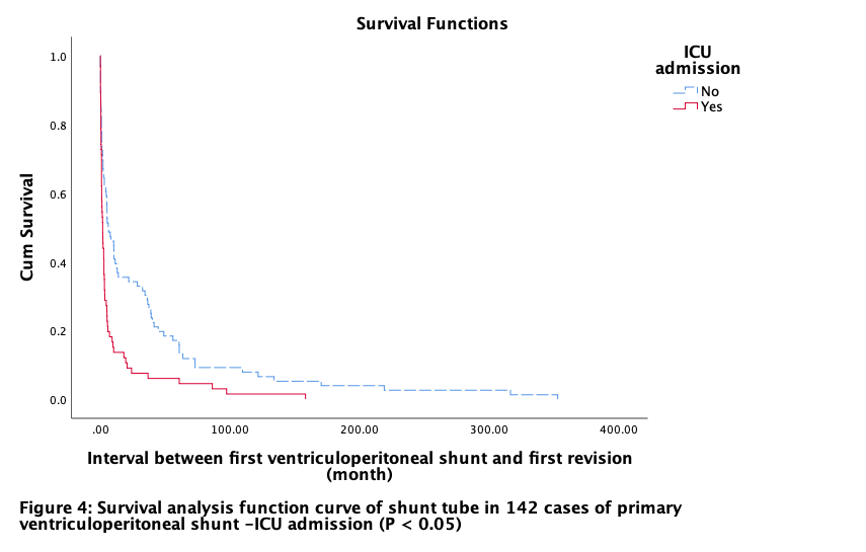


**Supplementary Figure 3.** ICU admission were grouped and analyzed by Kaplan-Meier survival (P < 0.05).


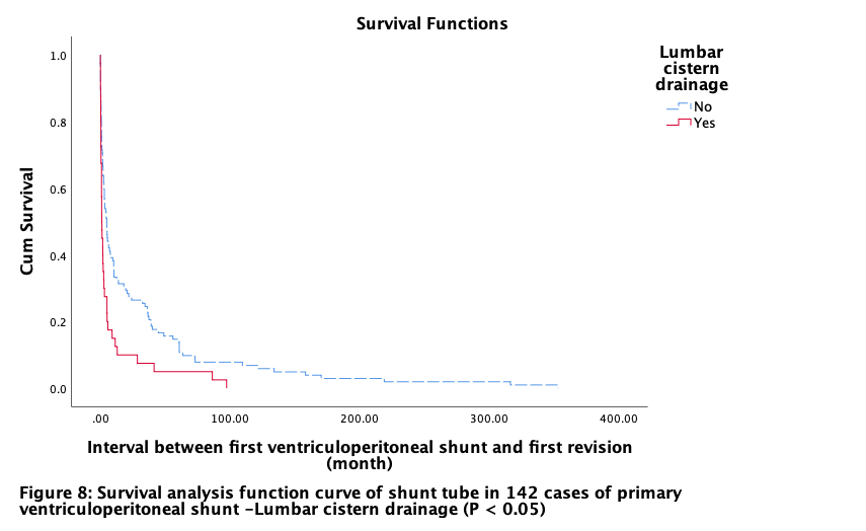


**Supplementary Figure 4.** Lumbar cistern drainage were grouped and analyzed by Kaplan-Meier survival (P < 0.05).


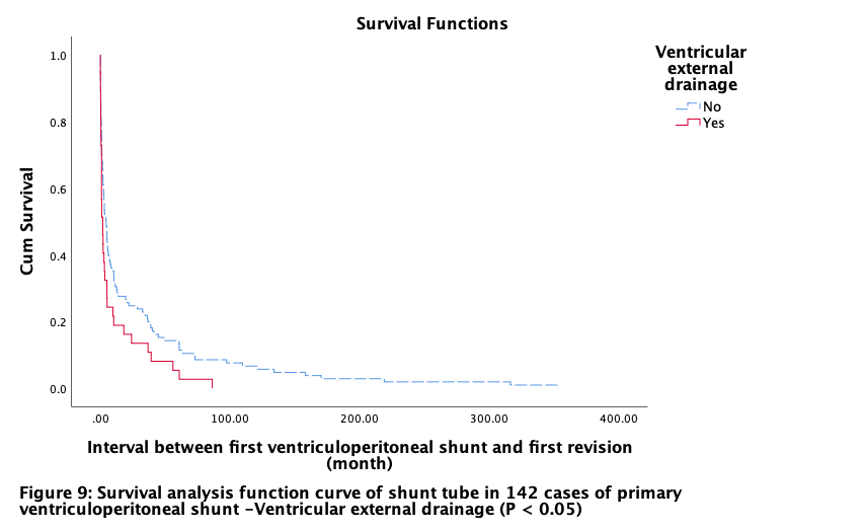


**Supplementary Figure 5.** Ventricular external drainage were grouped and analyzed by Kaplan-Meier survival (P < 0.05).
